# The introduction of a safe discharge network for the ongoing oral healthcare of patients who have completed management for head and neck cancers

**DOI:** 10.1038/s41415-025-9118-0

**Published:** 2026-01-09

**Authors:** Jaymit Patel, Zaid Ali

**Affiliations:** Consultant, Restorative Dentistry, Leeds Teaching Hospitals Trust, United Kingdom

## Abstract

Oral healthcare for patients who have been treated for head and neck cancer is essential to the long-term health and wellbeing of these patients. Access to routine dental care has, however, faced persistent challenges, resulting in a risk for oral health deterioration. We present the experience of West Yorkshire in developing collaborative shared care networks between a tertiary care centre and primary care dental services to safeguard oral health for this vulnerable population.

The methodologies used included needs assessments, pathway analyses and practitioner confidence surveys, alongside a range of health education initiatives.

We hope that sharing of these experiences will facilitate navigation of the structural and multi-organisational dimensions of healthcare planning and provision.

## Key points

- Highlights the importance of dental access for head and neck cancer patients.
- Summarises the challenges faced in improving dental access for this cohort.
- Describes an approach to improving dental access with multi-organisational collaboration.

## Background

Access to oral healthcare for the prevention, diagnosis and treatment of primary dental diseases and surveillance of oral health to detect more sinister pathologies is an important component of overall health, yet the landscape has been challenging for many years and continues to be of significant concern, particularly for vulnerable populations in the United Kingdom (UK). In the UK, patients who have undergone treatment for head and neck cancer (HNC) face a unique set of challenges in accessing dental services, as their particular clinical status as ‘clinically vulnerable' and susceptible to dental and recurrence of more sinister pathology is largely overlooked by the current iteration of the NHS (National Health Service) dental contracts for primary care, both in General Dental Services and Personal Dental Services. These challenges are compounded by the persistent disparities in healthcare access – health awareness and health outcomes associated with sociodemographic variables. Having a higher risk of HNC is ‘consistently associated with poor socioeconomic status' (SES) and with smoking.^1^^,^^2^

We know that SES plays a pivotal role in determining access to dental care and that individuals from lower socioeconomic backgrounds are more likely to experience barriers to care, including financial constraints and transportation issues.^3^ These barriers are exacerbated for patients with HNC, who may already face financial burdens due to financial burden associated with their cancer treatment and subsequent healthcare needs.^3^

Unsurprisingly, this background at the time of diagnosis results in the finding that patients who are diagnosed with HNC have poorer levels of baseline oral health when compared to the general populus: the percentage of patients with periodontal disease and carious teeth being greater in patients diagnosed with HNC.^4^

We summarise the West Yorkshire approach to improving oral health outcomes for patients treated for HNC.

## Impacts of HNC treatment on oral health

In addition to the poor levels of baseline oral health, patients who undergo treatment for HNC experience a substantial negative impact on their oral health-related quality of life, especially when radical radiotherapy is used as part of their treatment pathway.^5^ Patients experience a range of profound and lasting impacts on oral health and oral function, with issues including trismus, xerostomia, taste changes, dysphagia and osteoradionecrosis (ORN).^6^ These complications not only compromise oral function but can increase the incidence of dental caries, often referred to as radiation caries.^7^ Indeed, oral health is often an area of particular concern to patients after they have completed their curative treatments for HNC. The patient concerns inventory (PCI) is a valuable tool for identifying the specific concerns of patients. Patients often report significant worries related to their oral condition, including pain, difficulty chewing, and the aesthetic impact of oral changes on their appearance. These concerns highlight the psychosocial and social dimensions of oral health issues which may affect a person's ability to socialise, eat comfortably and maintain their self-esteem.^8^

**Fig. 1:**
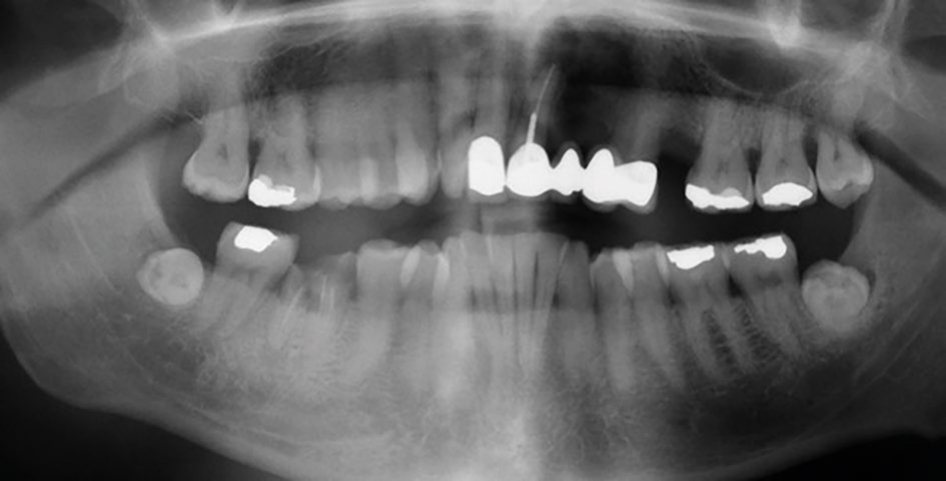
Dental panoramic radiograph of a patient before radiotherapy for head and neck cancer

**Fig. 2:**
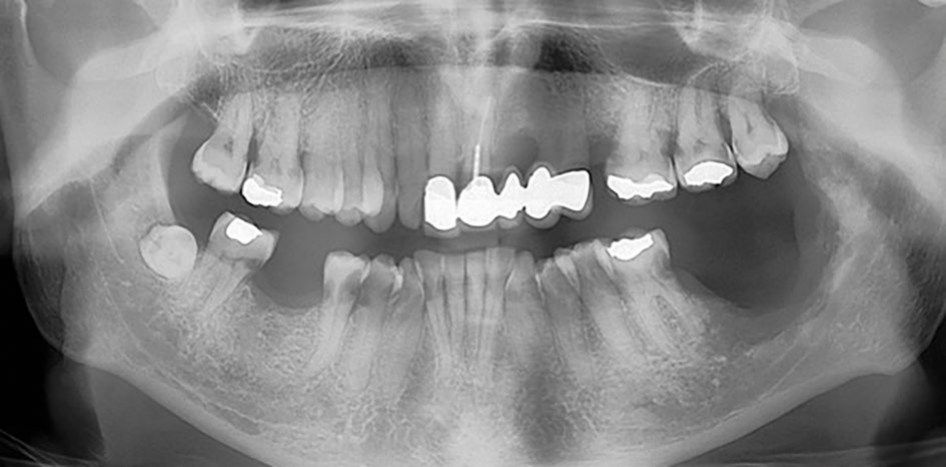
Dental panoramic radiograph for the same patient only 15 months after the completion of radiotherapy showing widespread radiation caries and osteoradionecrosis to the left posterior mandible

Consultant restorative dentists (CRDs) are a core aspect of the HNC treatment pathway, and their role aims to reduce the effects of pre-existing oral disease on a patient's experience of their HNC treatment pathway and to mitigate the effects of the HNC treatment pathway on longer-term oral health. CRDs are involved in the oversight and implementation of both primary dental disease stabilisation and complex oral and dental rehabilitation. The long-term success of these early interventions are, however, dependent on the patient's ability to access effective long-term supportive and maintenance care. Many HNC patients have a lifelong high risk for caries and for those who have experienced radical radiotherapy, the consequences of minor oral surgical procedures, such as a dental extraction, can be serious. ORN occurs in approximately 8% of HNC patients and is often associated with an extracted tooth in a zone of alveolar bone which has been irradiated. Both the experience of ORN and its treatment can be life-changing for the patient and costly to manage for both patients and health services. Figure 1 and Figure 2 demonstrate the deterioration of one HNC patient's dentition within less than 18 months of completing cancer treatment.

From an economic perspective, the consequences of poorer oral health following HNC treatment can be significant. Patients needing to undergo extensive dental care due to complications of their treatment often incur high healthcare costs, straining both personal finances for patients and the capacity of healthcare systems. At the Leeds Dental Institute, our recommended maintenance protocol for any patient having undergone radiotherapy as part of an HNC treatment regimen can be found in Box 1. This is informed by both national guidance and anecdotal experience.^9^^,^^10^^,^^11^

Box 1 Recommended maintenance protocol for primary care practitioners to follow when setting out maintenance arrangements for HNC patients treated with radiotherapy

- Recall at increased frequency, i.e., three-monthly for first year reducing to six-monthly if risk assessment supports this
- Diet advice
- Oral health instruction. This should be tailored to the patient and align with the RCSEng 2018 guidance
- Suggestion to use GC tooth mousse after evening meals. To apply to teeth with a soft brush, or to be used with a custom tray (fluoride tray)
- Fluoride:
  - Application of topical fluoride to exposed root dentine at recall visits
  - Prescription of high fluoride toothpaste (Duraphat 5,000 ppm)
  - Advice to use fluoride mouthwash twice daily
  - Fabrication of fluoride trays which should be used with either fluoride toothpaste or tooth mousse for up to 30 minutes several times per week
- Full mouth professional mechanical plaque removal at recall intervals, use local anaesthetic where pockets are >5 mm
- Restoration of new radiation caries as required using either composite resin or glass ionomer cement restorations
- Due to history of radiotherapy we recommend the avoidance of dental extractions in general practice due to the risk of osteoradionecrosis. Re-referral to the restorative team is recommended in cases where extractions are being considered

## Interactions between dental access and HNC

It is difficult to draw direct parallels or causative relationships between dental access, or the lack thereof, and experience of HNC. The interplay between these is complex and multifactorial. It is significantly confounded by variables such as socioeconomic deprivation, patient awareness, health education, lifestyle and access to diagnostic pathways. Socioeconomic deprivation is a critical determinant of health and of access to care. People living in areas of high deprivation can face substantial barriers to dental access and this lack of access can result in an exacerbation of existing oral health issues and worsening oral health.^12^ These factors may be the result of:^13^

- A limited number of dental practices/dentists working in deprived areas
- The ability of individuals with low SES to access dental care due to time/travel/cost barriers
- The willingness of individuals of low SES to access dental services due to the stigma, trust, fear or other barriers associated with dental care.

With respect to HNC, this delay to accessing care due to socioeconomically imposed barriers to care can clearly hinder timely diagnosis.^13^ We know that the stage at which HNC is diagnosed impacts significantly on survival, functional outcomes and quality-of-life outcomes for patients.^7^ Overall, ten-year survival for head and neck squamous cell carcinoma varies by site and in relation to the p16 status of the cancer (p16 being a marker for whether the cancer is associated with human papillomavirus). The worst overall survival is seen in p16-ve oropharynx (56%) and hypopharynx (51%) and the stage of cancer at diagnosis is found to significantly impact overall survival at ten-year follow-up.^14^ Later-stage cancers are more likely to require treatment involving multiple modalities and the nature of each to be more likely to involve sequelae, impacting on aesthetics, function and psychological wellbeing.^7^

A study investigating over 60,000 HNC referrals found that patients who were diagnosed through an emergency route were more likely to present with advanced disease.^15^ People diagnosed via this pathway were more likely to be from urban areas with higher levels of deprivation, from non-white ethnic groups, and over the age of 65. People being referred via ‘their dentist' were more likely to be women and those with oral cancer at a less advanced stage.^15^

## The challenges to improving dental access

Dental access remains a significant barrier to HNC patients. In addition to this, there is evidence that oral health is a concern for one-quarter of HNC patients after cancer treatment. Some studies suggest that the dentist is the most requested healthcare professional by HNC patients.^16^

Despite this, there is a lack of evidence investigating the reasons for dental access being such a challenge in HNC patients. There are multiple potential barriers to access, particularly in individuals with a low SES.^13^^,^^15^ An in-depth understanding of these challenges is essential to targeting access programmes.

**Fig. 3:**
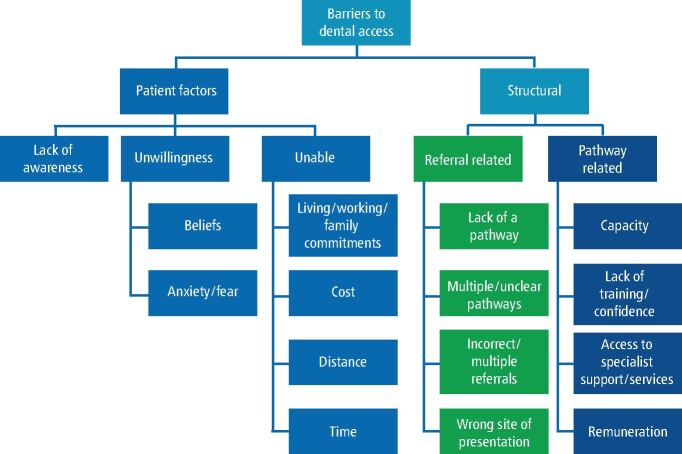
The potential barriers to head and neck cancer patients accessing routine dental care in primary dental services

The potential barriers to dental access for HNC patients are summarised in Figure 3.

Developing a safe discharge network (SDN) in West Yorkshire facilitated an improved understanding of the challenges to delivering care for HNC patients through a survey of dental practices in the West Yorkshire Local Dental Network (LDN). Delivery of dental care to HNC patients was discussed at an online practitioner engagement forum. The aim of this workshop was to identify key barriers to the delivery of care. The forum consisted of general dental practitioners during a LDN meeting. Practices were identified by a survey of dental practices in the region, which was led by the LDN chair. The LDN is a local network hosted by NHS England (NHSE), though its future remains unclear since the announcement in March 2025 of the abolition of NHSE. It consists of dental representatives, commissioners, public health officials, patient representatives, and NHS improvement leads. The process in West Yorkshire involved collaborative discussion to identify as many barriers to care as possible.

The survey consisted of four sections:

1. Practitioner information
2. Confidence in providing treatment types in non-case specific scenarios
3. Confidence in providing treatment in case-specific scenarios (five cases)
4. Practitioner support for interventions to improve confidence.

The survey was disseminated to 541 dental practices in Leeds, Yorkshire using a digital format (Microsoft Forms). All data were collected in an anonymous manner; however, respondents were given the opportunity to provide feedback or make queries via email. The contact details of these individuals were kept separate from the survey results, and all data were handled in accordance with information governance policies at Leeds Teaching Hospitals Trust and the Data Protection Act (2018). Individuals were provided with an introduction to the purpose of the survey and an estimate of the time required to complete the survey (11 minutes). Individuals were also provided with a survey completion deadline (30 days).

Data were collated in a Microsoft Excel spreadsheet and analysed using simple descriptive statistics.

Information from this initial phase informed the survey contents. All NHS practitioners registered with the West Yorkshire health service were provided with information on how to access the survey. This was disseminated by the commissioning health service via registered dental practice email addresses in line with accepted information governance principles and consent.

The survey identified three key themes as barriers to the provision of primary dental services for HNC patients:

1. Practitioner training and confidence
2. Remuneration
3. Access to specialist services.

The results of this process as summarised in an earlier publication.^17^ Further engagement sessions were held between dental practitioners who provided their contact information and the LDN. This discussion process explored methods to overcome barriers to access, while focusing on key aspirational outcomes for patients:

1. Guaranteed dental access at the point of delivery for all cancer patients (irrespective of their pre-existing access to dental services)
2. Guaranteed provision of care as per quality standards (Box 1).

The discussion process led to recruitment of eight dental practices across West Yorkshire in alignment with the above goals. Dental practitioners were supported with the delivery of care by provision of up to four times yearly education sessions on methods of care for, and overcome barriers to care for, HNC patients. Practitioners were also offered streamlined access to specialist advice, guidance and (where required) treatment via a referral pathway to the Leeds Dental Institute.

Following initiation of this pathway, one dental practice has left the network following sale of the practice; however, the network has expanded to include several services within the salaried Community Dental Services. A two-year survey of recruited practices has identified improvements in the proportion of respondents who were confident in the providing care for HNC patients with regard to:

- Examination and assessment
- Prevention
- Stabilisation of caries and periodontal disease, and composite bonding
- Endodontic treatments (including with inter-incisal opening <30 mm)
- Provision of fixed and removable prosthodontics.

The confidence of general dental practitioners in providing dental care for HNC patients is summarised in Table 1. In total, 60 individuals responded to the first survey (11% response rate) and 49 individuals responded to the second survey (9% response rate). All questions were answered and completed fully. The survey required a mean time to completion of 9 minutes and 23 seconds.

**Table 1:** Confidence of general dental practitioners in providing dental care for head and neck cancer patients

**Practitioner confidence**	**Percentage of respondents at baseline**	**Percentage of respondents at two years**
Extremely confident	9.50%	28.56%
Somewhat confident	36.05%	57.14%
Neutral	30.00%	0.00%
Somewhat not confident	24.45%	14.29%

These data highlight that while remuneration and the number of dental practices may be barriers to dental access, there are numerous other barriers to the provision of dental care that can be overcome through the formation of collaborative networks between primary care and specialist services.

Recent guidance published by NHSE further highlights the role of commissioned pathways to support the care of HNC patients.^18^ While this guidance documentation highlights the need for specific pathways, and the importance of safeguarding standards of care, it does not specify the structure of services. Indeed, it is likely that models of care will need to remain flexible between regions and over time to ensure that new and persistent barriers to care are addressed through collaborative action. This will be based on a number of factors, including:

- Pre-existing barriers to dental access
- The geographic distribution of patients
- Access to postgraduate-funded education
- Access to remuneration models (such as flexible commissioning).

## Reflections

The operation of a SDN for several years has afforded the team to reflect on the process – these reflections are summarised here.

The SDN was developed after multiple attempts to improve the long-term dental support available for HNC patients. Numerous different pathways were attempted but persistently failed to gain traction. One advantage of the present system is that it is pragmatic in that it demands minimal resources while providing most of the desirable features of a more complex healthcare network, such as a managed clinical network. In hindsight, it was beneficial to develop the network in a stepwise manner, starting with simple surveys and discussion of opportunities for collaboration, rather than a formal organisational network. This approach enabled a more nimble approach to service development in a manner that met the healthcare needs and challenges of West Yorkshire. Communication has remained pivotal throughout this process. As with any hub and spoke model, effective communication between primary care practitioners and hospital consultants is critical. This relates to the communication of overarching goals, as well as daily service operation. We focused on supporting practitioners and availing patients of effective and timely care.

An efficient communication method is essential and can be supported by regular meetings with practitioners, as well as digital referral/guidance systems. We recommend a hub and spoke model for most regions as this ensures widescale oversight of successes and failures. The tools used need not be complex – the majority of hub-to-spoke communication in the SDN is email-based. Having dedicated administrative time for the management of such as service is an important aspect of this.

As the network has developed from the inclusion of general dental practices to inclusion of Community Dental Services, we have found that it is important to remain adaptable and responsive to the needs of service providers. Our standard operating procedure and template discharge letters have evolved to include additional information as time has progressed and we have recently shared these with the Yorkshire Dental Clinical Leadership Group in the hope that NHSE will support the formalisation of this clinical network and support the widening of access to the network to a greater number of primary care practitioner groups.

Finally, it may worry some practitioners in primary care that they would be asked to support the care of patients who have high treatment and supportive needs and for whom routine dentistry may be more challenging than for the general populus. It is important therefore to reassure fellow colleagues that the approach in all instances is a patient-centred one. Some patients will have more dental rehabilitation within a tertiary referral centre, such as Leeds Dental Institute, to optimise their oral function and oral health-related quality of life. The requirement thereafter within primary care would remain focused on maintenance, prevention, and early detection of primary disease and stabilisation thereof. Of course, despite best efforts, deterioration in the oral condition may occur and at such a time, re-access to the tertiary centre would be indicated to re-visit further rehabilitation options and opportunities.
